# Cloud Computing-Based Framework for Breast Cancer Diagnosis Using Extreme Learning Machine

**DOI:** 10.3390/diagnostics11020241

**Published:** 2021-02-04

**Authors:** Vivek Lahoura, Harpreet Singh, Ashutosh Aggarwal, Bhisham Sharma, Mazin Abed Mohammed, Robertas Damaševičius, Seifedine Kadry, Korhan Cengiz

**Affiliations:** 1 Department of Computer Science and Engineering, DAV University, Jalandhar 144 012, Punjab, India; williamsvivek95@gmail.com (V.L.); harpreet99.nitj@gmail.com (H.S.); 2 Department of Computer Science and Engineering, Thapar Institute of Engineering and Technology, Patiala 147004, Punjab, India; er.ashutoshaggarwal@gmail.com; 3 Chitkara University School of Engineering and Technology, Chitkara University, Himachal Pradesh, India; bhisham.sharma@chitkarauniversity.edu.in; 4 Information Systems Department, College of Computer Science and Information Technology, University of Anbar, 55431 Ramadi, Anbar, Iraq; mazinalshujeary@uoanbar.edu.iq; 5 Department of Applied Informatics, Vytautas Magnus University, 44404 Kaunas, Lithuania; 6 Faculty of Applied Mathematics, Silesian University of Technology, 44-100 Gliwice, Poland; 7 Faculty of Applied Computing and Technology (FACT), Noroff University College, 4608 Kristiansand, Norway; skadry@gmail.com; 8 Department of Electrical—Electronics Engineering, Trakya University, Edirne 22030, Turkey; korhancengiz@trakya.edu.tr

**Keywords:** breast cancer, extreme learning machine, cloud computing, telehealth

## Abstract

Globally, breast cancer is one of the most significant causes of death among women. Early detection accompanied by prompt treatment can reduce the risk of death due to breast cancer. Currently, machine learning in cloud computing plays a pivotal role in disease diagnosis, but predominantly among the people living in remote areas where medical facilities are scarce. Diagnosis systems based on machine learning act as secondary readers and assist radiologists in the proper diagnosis of diseases, whereas cloud-based systems can support telehealth services and remote diagnostics. Techniques based on artificial neural networks (ANN) have attracted many researchers to explore their capability for disease diagnosis. Extreme learning machine (ELM) is one of the variants of ANN that has a huge potential for solving various classification problems. The framework proposed in this paper amalgamates three research domains: Firstly, ELM is applied for the diagnosis of breast cancer. Secondly, to eliminate insignificant features, the gain ratio feature selection method is employed. Lastly, a cloud computing-based system for remote diagnosis of breast cancer using ELM is proposed. The performance of the cloud-based ELM is compared with some state-of-the-art technologies for disease diagnosis. The results achieved on the Wisconsin Diagnostic Breast Cancer (WBCD) dataset indicate that the cloud-based ELM technique outperforms other results. The best performance results of ELM were found for both the standalone and cloud environments, which were compared. The important findings of the experimental results indicate that the accuracy achieved is 0.9868, the recall is 0.9130, the precision is 0.9054, and the F1-score is 0.8129.

## 1. Introduction

In recent decades, breast cancer has been a predominant cause of mortality amongst women [1,2]. Approximately 15% of mortalities among women are caused by breast cancer [3]. According to future projections of the World Health Organization (WHO), by 2040, the number of cases of breast cancer is predicted to reach 2.7 million worldwide [4]. The situation is alarming for many developing countries and has crippled medical facilities, where the medical staff have been overwhelmed by the COVID-19 pandemic. Early and accurate detection of breast cancer can lead to an early start in treatment and can increase the chances of survival [5], but it is difficult to diagnose cancer in the early stages, and therefore, techniques that can improve accurate detection of breast cancer are always solicited.

In the past few years, cloud computing [6] has emerged as a strong alternative to using costly locally managed computing resources. It is an on-demand service in which data can be processed and managed by storing it on the network of remote servers. Cloud computing services provide a browser-based dashboard, making it easier for the IT personnel to access the services provided by cloud service providers. Cloud computing is convenient for integrating data on the cloud, making it easier to update medical records. Moreover, cloud computing provides a large number of resources that can accommodate huge datasets of biomedical images or speech data [7]. A critical feature of cloud computing is the high availability of the services that can help healthcare industries provide uninterrupted services with less downtime [8]. 

Cloud computing services are essential for developing assisted living environments [9]. Furthermore, cloud computing services can be used to monitor patients, elderly people, and those with disabilities in remote or inaccessible villages and towns in many underdeveloped countries, where medical facilities and expertise are not readily available [10]. In these areas, women with breast cancer are often left undiagnosed, and ultimately, it is too late when they reach doctors available in larger cities. Doctors can use cloud computing to diagnose patients who cannot reach them due to a lack of financial resources. They can also use cloud computing for guidance through telehealth [11] and telemedicine [12], which includes the transmission of various medical data, such as high-resolution biomedical photographs and patient video recordings from remote areas to other geographic locations, where specialist physicians and large hospitals are situated. Cloud computing also enables essential services such as a rapid search tool for blood and organ donors in the case of emergencies [13].

Studies have investigated machine learning methods for increasing the accuracy of disease diagnoses [14,15,16,17] in addition to advanced medical imaging techniques such as mammography. One such method is extreme learning machine (ELM) [18], which is a kind of artificial neural network (ANN). ELM has been applied to various applications such as colorectal cancer [19]; thyroid disease [20]; Parkinson’s disease [21]; brain tumors [22]; osteoarthritis [23]; and most recently, COVID-19 pneumonia [24] diagnosis. Due to the advantages of fast learning speed and low computational cost, ELMs have become popular for solving many complex problems. The main contributions of this study are as follows:

- A design of a cloud-based diagnosis system to monitor remote user health data for breast cancer diagnosis is proposed. Through an analysis of consumer health data stored on cloud servers, the method is flexible enough to diagnose and classify a variety of diseases.
- ELM is used to classify patient data for breast cancer detection.
- The ELM model is compared with other traditional classification algorithms. Large datasets are supported using the cloud to reduce execution time; these classification models are compared using the cloud as well as a standalone platform.
- To further improve the model’s classification performance, feature selection is used to remove irrelevant features, and the hidden layer nodes of ELM are tuned.
- The best performance results of ELM for both standalone and cloud environments are compared.

The remainder of this paper is organized a follows: In Section 2, related work is presented; in Section 3, a description of the methodology used in this work is provided; in Section 4, we discuss the setup of the experimental environment; in Section 5, we discuss the various results obtained in this study; and, in Section 6, we discuss the implications of the results as well as the conclusions and future work.

## 2. Related Work

The diagnosis of breast cancer disease is an area of interest for many researchers [25]. Below, we discuss some of the disease diagnosis systems. Gupta et al. [26] proposed a heart disease prediction system using a cloud environment. Various algorithms including Random Forest (RF), J48, multi-layer perceptron, Naïve Bayes (NB), Binary Discriminant, Boosted tree, AdaBoost, and Support Vector Machine (SVM) were executed on the Cleveland dataset. Firstly, the algorithms were implemented on the standalone system by using various evaluation criteria. Later, the three algorithms that had the best accuracy were ensembled in the cloud environment. Saba et al. [27] discussed a framework in which breast cancer cells can be detected and classified using cytology images. Furthermore, features that incorporate shape were used to detect tumor cells using ANNs and an NB classifier. Goncalves et al. [28] discussed an approach to early breast cancer diagnosis. This work followed two different strategies. The first step involved in the classification process used ANN, and the second step focused on SVM. Rodriguez-Ruiz et al. [29] evaluated an Artificial Intelligent (AI) system against radiologists in the detection of breast cancer using digital mammograms. The results acquired proved that the AI system was able to detect breast cancer far more accurately than radiologists. Ragab et al. [30] suggested a system for the diagnosis of breast tumors. This system is segmented into two parts in which the features are retrieved using the deep convolutional network and support vector machines are used for obtaining better accuracy. Kashif et al. [31] suggested a hybrid model for predicting breast cancer from mammography images. First, the images were segmented and the features were extracted using mammogram processing and then classification was performed using the extracted features. 

Hamed et al. [32] proposed using the You Only Look Once (YOLO) and RetinaNet models for breast cancer recognition while achieving 91% accuracy of five mammogram image datasets. Ak [33] discussed various approaches of machine learning and applied them to the Wisconsin Diagnostic Breast Cancer (WBCD) dataset, focusing on comparative analysis and data visualization. Jeyanathan et al. [34] extracted features from breast thermograms using wavelet, curvelet, and contourlet transform for breast cancer recognition, achieving an accuracy of 91%, a sensitivity of 87%, and a specificity of 90% using the AdaBoost classifier. Abdar et al. [35] used voting and stacking techniques to construct a two-layer nested ensemble (NE) model with single classifiers (naïve Bayes and BayesNet), which was tested on the WDBC dataset, achieving an accuracy of 98.07%. Dhahri et al. [36] compared the performance of KNN, SVM, Decision Trees (DT), Random Forest (RF), AdaBoost, Gradient Boosting (GB), Gaussian Naïve Bayes (GNB), Linear Discriminant Analysis (LDA), quadratic discriminant analysis (QDA), linear regression, and extra trees classifier, while the features were selected using Genetic Programming (GP) optimization. The AdaBoost classifier seemed to exhibit the best accuracy of 98.24% on the WDBC dataset. Khan et al. [37] adopted pretrained CNNs (GoogLeNet, VGGNet, and ResNet), which were fed into a fully connected network layer for the classification of malignant and benign cells using average pooling classification, which achieved a 97.52% accuracy on two breast microscopic image datasets.

McKinney et al. [38] proposed an AI system that outperformed human experts in breast cancer prediction on mammogram images. Memon et al. [39] suggested using a modified recursive feature selection algorithm that achieved 99% accuracy with an SVM classifier on the WDBC dataset. Ronoud and Asadi [40] suggested using the genetic algorithm (GA) to evolve the number of hidden layers and neurons and to finetune the network weights and biases of the deep belief network (DBN). Finally, DBN was combined with an ELM classifier, which achieved 99.75% accuracy on the Breast Cancer Wisconsin—Original (WBCO) data and an accuracy of 99.12% on the WDBC dataset. Ting et al. [41] proposed a deep classification algorithm to detect and classify breast cancer in mammogram images, achieving an accuracy of 90.50% and a specificity of 90.71%. Vijayarajeswari et al. [42] combined Hough transform for feature extraction from mammograms and SVM for classification while achieving an accuracy of 94% on a small dataset of images. Wu et al. [43] suggested a deep CNN for breast cancer recognition, achieving an AUC of 0.89 over a large dataset of mammogram images. Assiri et al. [44] suggested using ensemble classification, which combined logistic regression learning, SVM with stochastic gradient descent optimization, and multilayer perceptron network, with a hard voting mechanism. This scheme achieved 99.42% accuracy on the WBCD dataset.

Table 1 presents a summary of some of the prominent disease diagnosis systems. Even though previous studies were promising, there is still room for improvement and development in breast cancer diagnosis methods. Some noteworthy facts used as inspiration are as follows:

1. Most of the studies did not consider feature selection and ELM as their primary algorithm for the diagnosis of breast cancer.
2. The most important issue is that many of the previous studies restricted their models to standalone systems, and thus, they are not available anytime and anywhere.
3. Many of these studies are unique to a particular field of study, but the approach should apply to all fields.

To solve the above issues, this study contributes to the following points:

1. ELM is considered as the primary classification algorithm.
2. To further improve the model’s classification performance, feature selection is used and the hidden layer nodes of ELM are tuned.
3. The ELM model is deployed in the cloud environment.

## 3. Cloud-Based Breast Cancer Diagnosis Model

**Figure 1:**
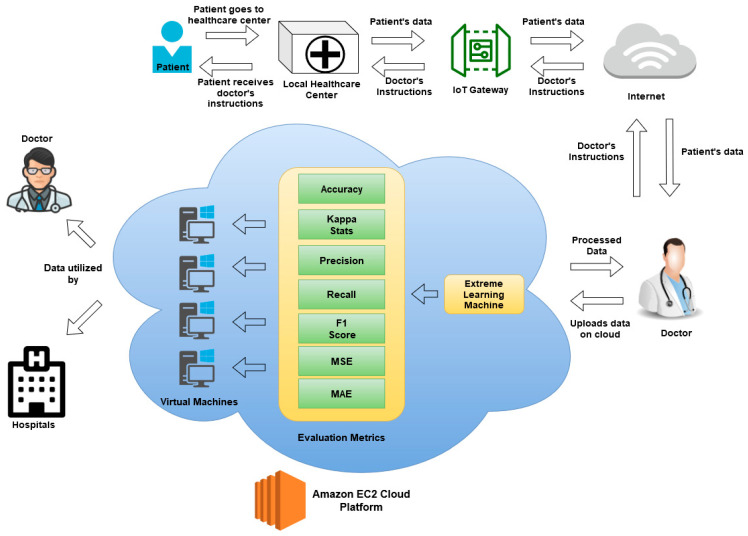
Components of the proposed architecture.

This research proposes the design of a cloud-based breast cancer diagnosis system that provides monitoring of remote user health data for the identification of breast cancer. When analyzing consumer health data stored on cloud servers, the method is flexible enough to diagnose and classify a variety of diseases. However, in this paper, we concentrated primarily on only one case of usage, namely defining the disease as “cancerous” or “noncancerous”. The outline of our proposed architecture is shown in Figure 1. In the proposed architecture, the patient goes to a remote healthcare center in their village, where the healthcare service provider collects the data from the patient, such as x-rays and other health parameters, and sends the data via the Internet to a doctor; then, the doctor uploads the data to the cloud platform for further processing.

In the cloud, the processing occurs in two stages. It has been established by past researchers that attribute selection improves the performance of machine learning methods [56,57,58]. Therefore, in the first stage, the gain ratio method is used to identify the significant features and to then remove the insignificant ones. The purpose of this step is to reduce the computational complexity. In the second stage, the classification is applied using ELM. 

### 3.1. Gain Ratio

The gain ratio [59] is a single attribute evaluation method that uses ranking to eliminate irrelevant attributes. It improves the information gain method by removing the favoritism towards attributes with many values by normalizing the formula of information gain using the information value of the split. It is a filter method that performs noniterative computation on the dataset to find the relevance of the attribute by using the following formula:

(1) $$Gain\;ratio\;\left(X\right)=\frac{Information\;Gain\;\left(X\right)}{H\left(X\right)},$$

where $H\left(X\right)=\;\sum_{i}-P_{j}\;\log_{2}\;P_{j}$, where $P_{j}$ is the probability of having a class *j*.

### 3.2. Extreme Learning Machine (ELM)

**Figure 2:**
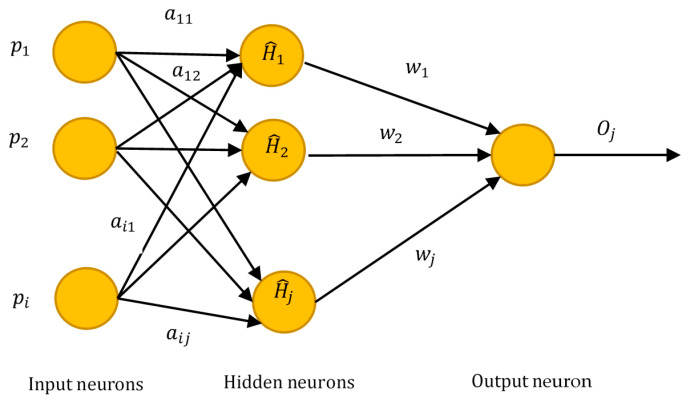
Extreme learning machine.

ELM [60] is a type of feed-forward neural network usually used for classification, regression, clustering, small estimate, compression, and pattern learning with either a single layer or various layers of hidden nodes, where the parameters of hidden nodes that include biases and weights need not be adjusted. On the other hand, the parameters of hidden nodes can be allocated randomly and never changed or can be inherited from their ancestors without alteration. These models learn extremely quicker than networks trained with backpropagation. The prevalent learning procedure used in feed-forward neural networks is the learning procedure for backpropagation, where propagating from the output to the input gradients can be determined. However, backpropagation possesses many problems. The training process is very time-consuming in most applications as weights and biases are rationalized afterward each iteration. To achieve maximum accuracy, the weight magnitude is disregarded in this model, due to which the output becomes worse over time. The local minima also affect the efficiency of the learning algorithm for backpropagation. ELM is a feed-forward network that removes the barrier of updating weights and biases. It focuses not only on minimum training error but also on achieving the lowest weight standards that increase the overall efficiency of this model. The problem of trapping in local minima is handled using simple alternatives avoiding such trivial issues. Figure 2 presents the working of ELM.

For *H* arbitrary samples $\left(p_{i},\;t_{i}\right)$, where $p_{i}={\left[p_{i1},\;p_{i2}\ldots \ldots .p_{in}\right]}^{T}\in \;Q^{n}$ and $\;t_{i}={\left[t_{i1},\;t_{i2}\ldots \ldots .t_{im}\right]}^{T}\in \;Q^{m}$, the standard single-hidden layer feedforward neural networks (SLFNs) with activation function f(·) and *G* hidden nodes can be written as

(2) $$\sum_{i=1}^{G}w_{i}f_{i}\left(p_{j}\right)=\sum_{i=1}^{G}w_{i}f(a_{i}\times \;p_{j}+c_{i})=\;o_{j},\;\left(j=1,2,\;\ldots H\right),$$

where $a_{i}={\left[a_{i1},\;a_{i2}\ldots \ldots .a_{in}\right]}^{T}$ is the weight vector linking *i*th hidden node and input nodes,$\;w_{i}={\left[w_{i1},\;w_{i2}\ldots \ldots .w_{in}\right]}^{T}\;$ is the weight vector linking *i*th hidden node to output node, $c_{i}$ is the threshold of hidden node, and $o_{j}={\left[o_{j1},\;o_{j2}\ldots \ldots .o_{jm}\right]}^{T}$ is the *j*th output vector of SLFNs.

Standard SLFNs with *G* hidden nodes and activation function f(·) can estimate these *H* illustrations with zero error, which means that $\sum_{j=1}^{G}\left|\left|o_{j}-t_{j}\right|\right|=0$ and that there exist ω_*i*_, $a_{i}$, and $c_{i}$ such that

(3) $$\sum_{j=1}^{G}w_{i}f(a_{i}\times y_{j}+c_{i})=t_{j}\;\left(j=1,2,\ldots H\right),$$

The above equation can be summarized as follows:

(4) Mw=T,

where

(5) $$M\left(a_{1},\ldots ,a_{G},\;c_{1},\ldots ,\;c_{G},\;y_{1},\ldots ,\;y_{G}\right)=\;{\left[\begin{array}{ccc} f\left(a_{1}\times y_{1}+c_{1}\right) & \cdots & f\left(a_{G}\times y_{1}+c_{G}\right)\\ \vdots & \cdots & \vdots \\ f\left(a_{1}\times y_{H}+c_{1}\right) & \cdots & f\left(a_{G}\times y_{H}+c_{G}\right) \end{array}\right]}_{H\;\times \;G},$$

(6) $$w={\left[\begin{array}{c} w_{1}^{T}\\ .\\ .\\ .\\ w_{Ň}^{T} \end{array}\right]}_{G\;\times \;n},$$

(7) $$T={\left[\begin{array}{c} t_{1}^{T}\\ .\\ .\\ .\\ t_{N}^{T} \end{array}\right]}_{G\;\times \;n},$$

where *M* is called an output matrix of hidden layer and the *k*th column of *M* is the output of the *k*th hidden node according to inputs $y_{1},\;y_{2}\ldots \ldots .y_{H}$. The solution of the linear system is

(8) $$w=\;M^{-1}T,$$

where *M*^−1^ is the Moore–Penrose generalized inverse of matrix *M*. 

The output function of ELM is defined as

(9) $$g\left(y\right)=p\left(y\right)w=p\left(y\right)\;M^{-1}T,$$

In ELM training, there are three key parameters. These are training set $K=\{\left(y_{j},\;t_{j}\right)$| $y_{j}\in Q^{n}$, $t_{j}\;\in \;Q^{m}$, j=1, ….. H}; the hidden node output function $f\left(a_{i},\;c_{i},\;y_{j}\right)$; and the hidden node number *G*. Once the values of the parameters are set properly, the training process of ELM can be initiated. Firstly, ELM randomly generates values for the *G* pair of hidden nodes parameters $\left(a_{i},\;c_{i}\right)$. Then, the output matrix *M* is generated using Equation (4) according to the input and arbitrarily produced parameters. Then, the output weight vector ω is generated using Equation (8). The classification outcome of test data tuples can be forecasted using Equation (9) after the training phase is completed. 

The training of ELM is performed as follows. 

Input a training set $A=\{\left(a_{i},\;d_{i}\right)|a_{i}\in \;X_{n},\;d_{i}\in \;X_{m},\;i=1,\ldots .,N\}$, activation function f(x), and number of hidden neurons *N*.

1. The weights *w_i_* of input and bias *b_i_* are allocated randomly.
2. The output matrix *M* of the hidden layer is computed.
3. Compute the output weight *w* as

(10) w=M×T,

where *M* and *T* are represented by Equations (4) and (5), respectively.

### 3.3. Evaluation Criteria

The key idea of this study is to diagnose an input sample whether it belongs to a class of positive samples or belongs to negative samples. There are four possibilities of prediction, which can be described using the terms shown in Table 2. 

Further, Table 3 shows the formulae of evaluation metrics. Classification accuracy is the total number of data tuples correctly classified out of the total number of classifications. Precision is the number of positive outcomes correctly classified out of the total positive outcomes forecasted by the classifier. Recall is the proportion of correct predictions of positives to the total number of actual positives. Kappa is a helpful evaluation metric, but due to its complexities, it is underutilized. This metric helps in problems in which there is multiclass classification. F-score represents the harmonic mean between precision and recall with values falling in [0, 1]. It shows the accuracy and reliability of the classifier. 

## 4. Research Materials and Methods

**Figure 3:**
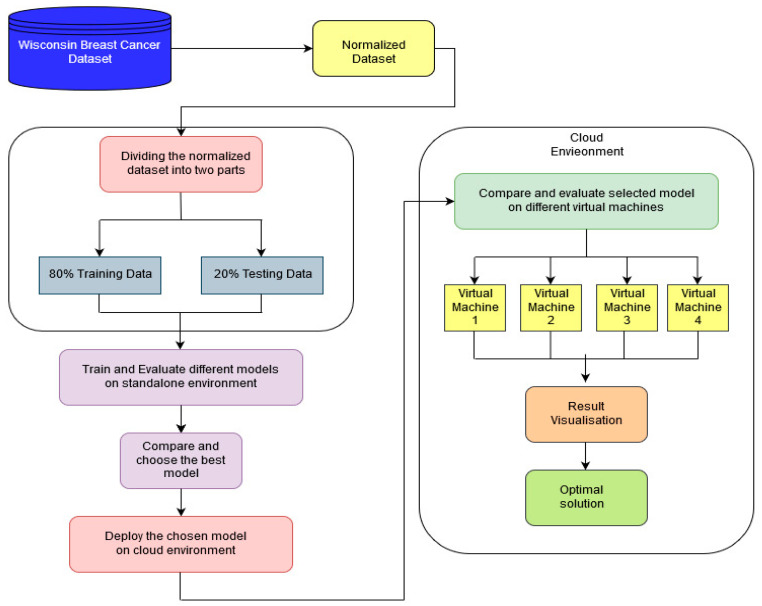
Experimental steps.

The experimental methodology of this study is divided into two parts. Firstly, the authors considered multiple classification models that include K-nearest neighbors [61], Naïve Bayes [62], Perceptron network [63], AdaBoost [64], and Support Vector Machine [65] and compared all these classification models with the ELM on the standalone environment, and later, the ELM model was deployed on the cloud environment. Firstly, the parameters of ELM were varied, and among those, the best results were determined. Later, the best model of ELM was compared with different classification models. The experimental steps are shown in Figure 3. Both the standalone and cloud computing environments are discussed below. 

### 4.1. Cloud Environment

In the cloud environment, Platform-as-a-Service (PaaS) was used on the Amazon EC2 (Amazon.com, Inc., Seattle, Washington, DC, USA) cloud to deploy the ELM models that were compared on the standalone system. The main reason for deploying the models on the cloud environment was to decrease the execution time and to increase the accuracy. Furthermore, shifting the models on the cloud also helps the models remain readily available anytime and anywhere. The virtual machines that have been used in the cloud environment are all based on a LINUX operating system. Later, the results from both the cloud environment and standalone system were compared. 

This process was also deployed on the Amazon EC2 cloud environment. The operating system of the virtual machine that was used on the cloud platform was Ubuntu (Canonical Ltd., London, United Kingdom), and the other parameters such as the number of CPUs, HDD space, and RAM varied. The instances m4.xlarge and c5.xlarge were utilized, which have an Intel Xeon (Intel Corporation, Santa Clara, CA, USA) processor. The feature subset selection was performed using Weka ver. 3.8 (University of Waikato, New Zealand) [66].

### 4.2. Standalone Environment

The standalone system that was used to carry out the experimentation had the following hardware configuration: (1) a memory of 8 GB, (2) Intel i5-7200 u (Intel Corporation, Santa Clara, CA, USA) with a base clock speed of 2.71 GHz, and (3) an HDD space of 1 TB. In this environment, various classification models were implemented using PyCharm IDE ver. 2020.2 (JetBrains s.r.o., Prague, Czech Republic) [67] on the WBCD dataset and were evaluated using various evaluation metrics. 

### 4.3. Collection of Data

The Wisconsin Breast Cancer Diagnosis (WBCD) [68] dataset was used for the experiment. The dataset consisted of 569 entries and 32 attributes, with the diagnosis attribute signifying malignant or benign. Table 4 shows the description of the 32 attributes.

To find the subset of relevant attributes for the classification process using the gain ratio method, ELM with 100 hidden nodes was used. The gain ratio ranked the attributes according to their relevance. To find the final subset of attributes, the accuracy of ELM was found with the first *n* attributes, where *n* varied from 3 to 32, and it was observed that the first 14 attributes in that rank list gave the maximum accuracy. In the order of rank, the serial number of these attributes were 24, 22, 25, 29, 9, 8, 28, 4, 5, 15, 7, 12, 14, and 27; hence, these 14 attributes were selected for further experiments.

## 5. Results

This section contains the results that were collected from both the standalone and cloud environments, and the results were compared to visualize the performance as we shifted from the standalone environment to the cloud environment. 

### 5.1. Performance Analysis on Standalone Environment

First, AdaBoost, SVM, naïve Bayesian, perceptron, and KNN and, then, the ELM model were executed on a standalone environment.

#### 5.1.1. Performance Analysis of ELM with Different Hidden Nodes

**Figure 4:**
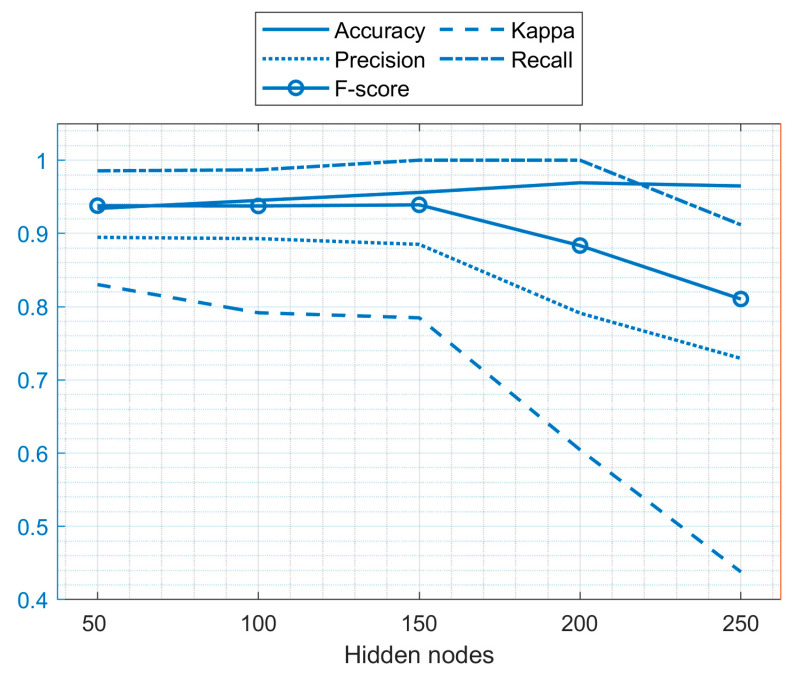
Graphical representation of the ELM performance (standalone environment).

An attempt was made by the authors to improve the accuracy of ELM by altering the number of nodes in the hidden layer in the ELM model. The minimum hidden layer node count was 50, and the maximum was 250. It was observed that ELM had a performance boost and gave better results when the hidden layer nodes count was altered. Table 5 summarizes the results, while Figure 4 presents a visual illustration.

It is clear from Table 5 that the ELM has maximum accuracy when the number of hidden layer nodes is 200, followed by the number of hidden layer nodes set at 250, 150, 100, and 50. Although the Kappa value when the number of hidden layer nodes is 200 is lower compared to when there are 50, 100, and 150 hidden layer nodes, it can be observed that the recall and F-score values for the 200 and 150 hidden layer nodes are higher compared to the values for the other hidden layer nodes. Hence, from the above comparison using various metrics, it can be observed that the ELM model gives the best accuracy (0.969) when the number of hidden layer nodes is taken as 200, followed by 250 (0.9648), 150 (0.956), 100 (0.945), and 50 (0.934). As the number of nodes increases in the hidden layers, each input is handled with multiple neurons, which reduces the load on a single neuron making computation less complex, but as the number of nodes exceeds a certain limit, the computation splits up among different neurons, making it more complex to handle the inputs, ultimately reducing the overall performance of the model [69].

#### 5.1.2. Performance Comparison of ELM with Various Classification Models

**Figure 5:**
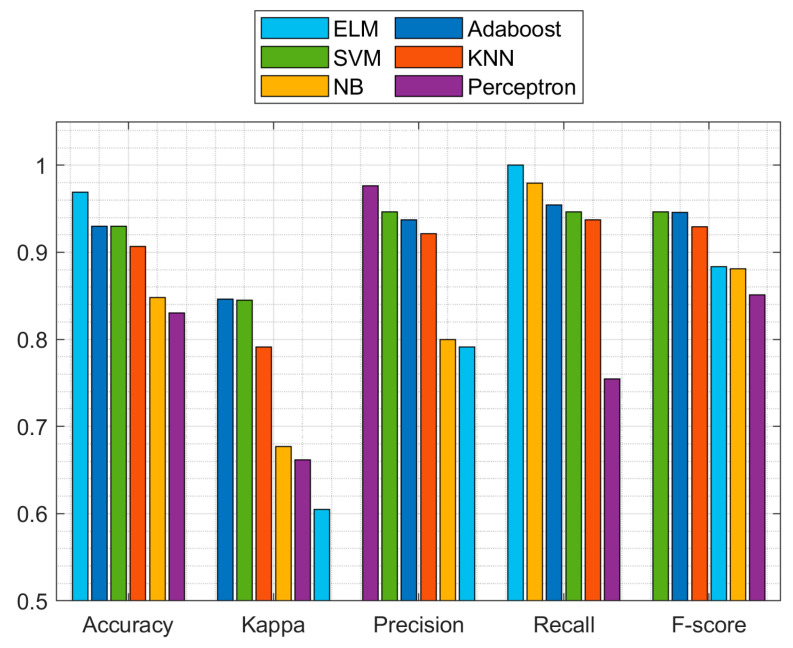
Graphical representation of the model performance (standalone environment).

In this section, the performance of various classifiers is compared with the ELM classifier with 200 nodes in a standalone environment. Note that 80% of the tuples in the dataset are used for training and the rest of the 20% tuples are used for testing. Metrics of evaluation such as Kappa statistics, accuracy, precision, recall, and F-score were used for comparison. Table 6 summarizes the results, while Figure 5 presents a visual illustration. 

From Table 6, by comparing the values of accuracy, ELM provides the highest accuracy of 0.9692 whereas perceptron provides the lowest accuracy of 0.8304. Moreover, ELM has the best recall value (1.00) among all the other classifiers. From the results shown above, it is observed that the ELM-based model provides the highest accuracy of classification followed by SVM, KNN, naïve Bayes (NB), AdaBoost, and perceptron.

### 5.2. Performance Analysis on Cloud Environment (Amazon EC2)

After comparing the various models in a standalone environment, we observed that the ELM outperformed all the other traditional classification procedures. Therefore, the ELM model was deployed on the cloud environment on virtual machines with different configurations.

#### Analysis of ELM Performance Using Different Hidden Layer Nodes

As observed, varying the hidden layer node number in the ELM increased its efficiency in the standalone environment. Therefore, the same procedure was followed in the cloud environment to boost the performance of ELM by varying the number of nodes in the hidden layer of the ELM model. Table 7 presents the results obtained on different virtual CPUs (vCPUs) and RAM. Below are the results that were obtained in the cloud environment.

**Figure 6:**
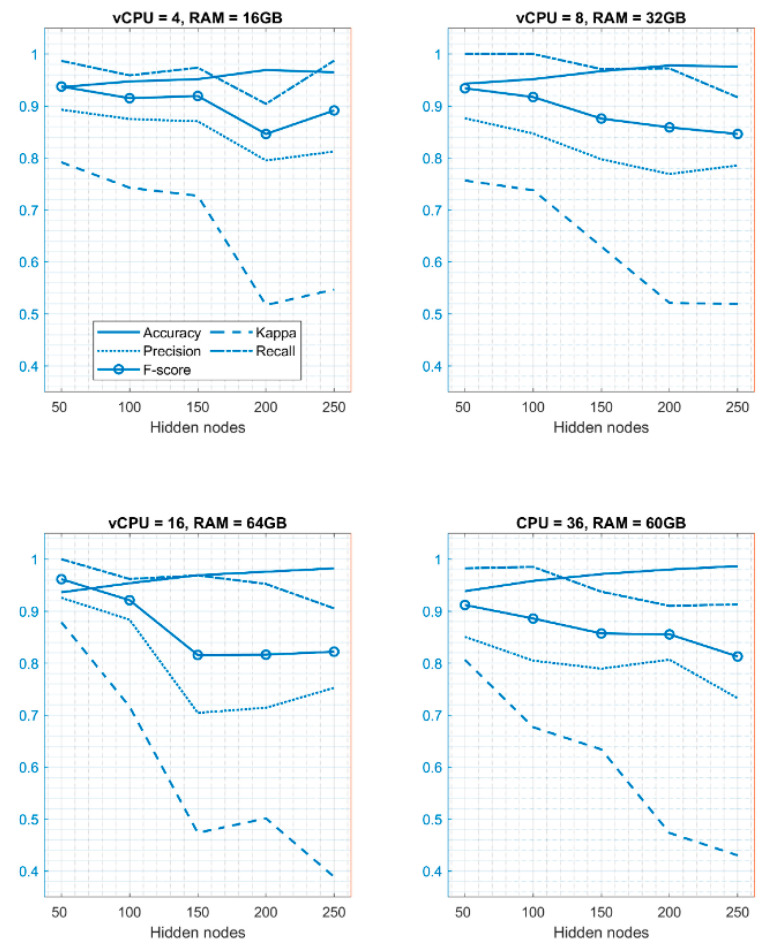
Graphical representation of the ELM model performance (cloud computing environment).

From Table 7, it can be observed that, when the number of hidden layer nodes is set to 200, ELM gives a better performance when there are 4 vCPUs and 16 GB RAM, and the same is the case when there are 8 vCPUs and 32 GB RAM. However, there is an increase in performance when the number of hidden layer nodes is set to 250 and when the number of vCPUs is 16 and RAM is 64 GB, and ELM obtains the best accuracy of 0.9868 when the number of vCPUs is 36 and RAM is 60 GB. It can be seen that, as the number of virtual CPUs, RAM, and number of hidden layer nodes increase, the classification accuracy increases. It can therefore be seen from the above comparison that ELM provides the best classification accuracy of 0.9868 based on different metrics when the number of hidden layer nodes is set to 250 and when vCPU is 36 and RAM is 60 GB. Hence, the ELM model with 250 hidden layer nodes implemented on a virtual machine with 36 vCPUs and 60 GB of RAM is considered best for the classification of breast cancer. Finally, the results are visualized in Figure 6.

### 5.3. Performance Comparison of ELM on the Cloud Environment and Standalone Environment

The best performance results of ELM were taken for both the standalone and cloud environment, and a comparison was made.

**Figure 7:**
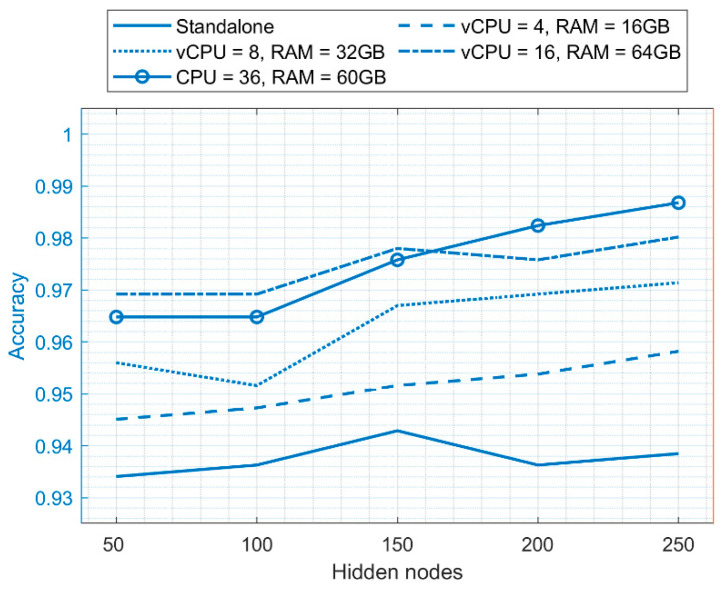
Comparison of the accuracy achieved when using standalone and cloud computing environments.

Figure 7 shows ELM’s success in the standalone and cloud environments with specific hidden layer nodes. The classification accuracy of ELM with 250 hidden layers nodes in a standalone environment is 0.9648, whereas in a cloud environment, the classification accuracy is 0.9868 when the number of vCPUs is 36 and RAM is 60 GB. This proves that ELM has a better classification accuracy in the cloud environment when diagnosing patients, whether they are suffering from breast cancer or not, utilizing the data provided by them.

For execution time, there is an improvement of about 18% (from 3.35 s on the standalone computer to 2.81 s on the cloud environment) when we deployed the model to the cloud environment. ELM has a shorter training time since it is a single-layer network feedforward. When a model is deployed on a cloud environment, the execution time is further reduced as resources are available in bulk and allows for computation in a shorter time.

One of the prominent causes of mortality among women is breast cancer. Early determination of this cancer increases survival chances, but women residing in medically underserved areas do not have access to specialist doctors. Machine learning and cloud computing services have drawn the attention of various researchers for developing disease prediction systems, such as [70,71,72,73,74,75,76,77,78], as a feasible option in remote diagnostics, where cloud computing provided Platform-as-a-Service (PaaS) to obtain resources on demand. 

## 6. Conclusions

This paper proposed a framework for cloud-based breast cancer diagnosis using Extreme Learning Machine (ELM) as a classifier. Cloud computing can provide unceasing services anytime and anywhere, which is beneficial for the healthcare industry as they can access the system whenever they want. Moreover, the cloud environment also provides resources that improve the overall classification accuracy of the proposed model. The main advantage of ELM is that the parameters such as weights and biases need not be adjusted, which makes it extremely faster and simpler than all the other gradient-based learning algorithms. In this context, this study proposed a cloud-based architecture for the diagnosis of breast cancer, which collected the data of the patient at remote healthcare centers established near villages and sent the data using cloud services to specialist doctors for analysis and for the provision of further instructions to the patients. 

We implemented various classifiers on the WBCD dataset for the diagnosis of breast cancer. Firstly, the gain ratio method was used to select the most relevant attributes and to discard irrelevant attributes. Secondly, various state-of-the-art algorithms were applied and compared with ELM on the standalone system. Further, the ELM model was deployed on the cloud environment using the Amazon EC2 cloud platform. ELM models with different hidden layer nodes were compared on the cloud environment, and the results obtained during experimentation validated that the accuracy of ELM increased in the cloud environment. Therefore, in that case, cloud computing will provide a stable platform since it provides higher accuracy and less execution time than the standalone platform. 

The performance of the cloud-based ELM was compared with some state-of-the-art technologies for disease diagnosis. The results achieved on the Wisconsin Diagnostic Breast Cancer (WBCD) dataset indicate that the cloud-based ELM technique outperforms other techniques. The best performance results of ELM were taken from both the standalone and cloud environments, and a comparison has been made. The findings of the experimental results indicate that the accuracy achieved is 0.9868, the recall is 0.9130, the precision is 0.9054, and the F1-score is 0.8129.

In the future, this framework can be further extended by using more resources in a cloud environment that may further increase the classification accuracy of the proposed framework. Moreover, various parameters of ELM can be tuned to further increase the performance of the proposed framework, and the proposed model can also be implemented in the area of image processing, under which various applications such as character recognition, medical imaging, satellite imagery, and photograph enhancement can be implemented.

## Figures and Tables

**Table 1:** Summary of related work on disease diagnostics.

Authors	Disease	Year	Dataset	Classifier	Accuracy (Highest)
Li et al. [45]	Thyroid	2012	Thyroid database from UCI repository	PCA-ELM	PCA-ELM = 98.1%
Sartakhti et al. [46]	Hepatitis disease	2012	hepatitis B dataset UCI Repository	SVM-SA	SVM-SA = 96.2%
Kumari et al. [47]	Diabetes	2013	Pima Indian diabetes dataset,	SVM	SVM = 78%
Kaya et al. [48]	Hepatitis disease	2013	hepatitis B dataset from UCI Repository	Rough Set ELM	Test/Train split 80/20 = 100%
Wang et al. [49]	Breast Cancer	2014	482 mammographs	ELM, SVM	ELM = 83%
Zheng et al. [50]	Breast Cancer	2014	Breast Cancer Wisconsin Dataset (BCWD)	K-SVM	K-SVM = 97.38%
Prashanth et al. [51]	Parkinson’s Disease	2016	PPMI database	Naïve Bayes, LR, Boosted Tree Random Forest, SVM	Random Forest = 96.18%
Chen et al. [52]	Parkinson’s disease	2016	PD dataset from UCI repository	ELM K-ELM	Accuracy = 96.47%
Esteva et al. [53]	Skin Cancer	2017	129,450 clinical images,	Deep CNN	CNN = 72.1%
Liu et al. [54]	Prostate Cancer	2017	341 cases	XMasNet (Based on CNN)	XMasNet = 84%
Chen et al. [55]	Disease Prediction	2017	31,919 hospitalized	CNN-MDRP	CNN-MDRP = 94.8%

**Table 2:** Four cases concerning the prediction.

Case	Definition
True Positive (TP)	A model forecasts the positive class correctly.
True Negative (TN)	A model forecasts the negative class correctly.
False Positive (FP)	A model forecasts the positive class correctly.
False Negative (FN)	A model forecasts the negative class incorrectly.

**Table 3:** Evaluation metrics of classification performance.

Formula	Expected Value
$Accuracy=\frac{\left(TP+FN\right)}{Total\;number\;of\;samples}$	High
Precision=TP/(TP+FP)	High
Recall=TP/(TP+FN)	High
$Kappa=\frac{P_{0}-P_{a}}{1-P_{a}}$	Value = 1 implies perfect agreement, and Value < 1 implies a less perfect agreement
$F-Score=\frac{2\times Recall\times Precision}{\left(Recall+Precision\right)}$	Best Value is 1, and Worst Value is 0

**Note**—*P*_0_ is the observed agreement, and *P_a_* is the expected agreement: $y_{j}$ is the anticipated output, and ${\hat{y}}_{j}$ is the predicted value.

**Table 4:** Dataset description.

S. No.	Attribute Name	Description
1.	Id	Id Number
2.	Diagnosis	The diagnosis of breast tissues (M = malignant, B = Benign)
3.	Radius_Mean	Mean of distances from the center to points on the perimeter
4.	Texture_Mean	Standard deviation of grayscale values
5.	Perimeter_Mean	Mean size of the core tumor
6.	Area_Mean	Mean area of the core tumor
7.	Smoothness_Mean	Mean of local variation in radius lengths
8.	Compactness_Mean	Mean of *perimeter*^2^/*area* − 1
9.	Concavity_Mean	Mean of severity of concave portion of the contour
10.	Concave points_mean	Mean for number of concave portions of the contour
11.	Symmetry_mean	
12.	Fractal_dimension_mean	Mean for *coastline approximation* − 1
13.	Radius_se	Standard error for the mean of distances from the center to the points on the perimeter
14.	Texture_se	Standard error for standard deviation for grayscale values
15.	Perimeter_se	
16.	Area_se	
17.	Smoothness_se	Standard error for local variation in radius lengths
18.	Compactness_se	Standard error for *perimeter*^2^/*area* − 1
19.	Concavity_se	Standard error for severity of concave portions of the contour
20.	Concave points_se	Standard error for the number of concave portions of the contour
21.	Symmetry_se	
22.	Fractal_dimension_se	Standard error for *coastline approximation* − 1
23.	Radius_worst	“worst” or largest mean value for the mean of distances from the center to points on perimeter
24.	Texture_worst	“worst” or largest mean value for standard deviation of grayscale values
25.	Perimeter_worst	
26.	Area_worst	
27.	Smoothness_worst	“worst” or largest mean value for local variation in radius length
28.	Compactness_worst	“worst” or largest mean value for *perimeter*^2^/*area* − 1
29.	Concavity_worst	“worst” or largest mean value for severity of concave portions of the contour
30.	Concave points_worst	“worst” or largest mean value for number of concave portions of the contour
31.	Symmetry_worst	
32.	Fractal_dimension_worst	“worst” or largest mean value for *coastline approximation* − 1

**Table 5:** Evaluation metrics for extreme learning machine (ELM) with different hidden layer nodes in the standalone environment.

Nodes in the Hidden Layer	50	100	150	200	250
Accuracy	0.9341	0.9451	0.9560	0.9692	0.9648
Kappa	0.8302	0.7917	0.7848	0.6046	0.4379
Precision	0.8947	0.8929	0.8851	0.7912	0.7294
Recall	0.9855	0.9868	1.0	1.0	0.9118
F-score	0.9379	0.9375	0.9390	0.8834	0.8105

**Table 6:** Performance of different machine learning models deployed in a standalone environment.

	AdaBoost	KNN	NB	Perceptron	SVM	ELM
Accuracy	0.9298	0.9064	0.8480	0.8304	0.9298	0.9692
Kappa	0.8460	0.7913	0.6768	0.6614	0.8447	0.6046
Precision	0.9375	0.9211	0.8000	0.9765	0.9464	0.7912
Recall	0.9545	0.9375	0.9796	0.7545	0.9464	1.000
F-score	0.9459	0.9292	0.8807	0.8513	0.9464	0.8834

**Table 7:** Performance analysis of ELM deployed on a cloud computing environment with different numbers of hidden layer nodes. Best accuracy values are shown in bold.

vCPU = 4 RAM = 16 GB	ELM (50)	ELM (100)	ELM (150)	ELM (200)	ELM (250)
Accuracy	0.9363	0.9473	0.9516	**0.9692**	0.9648
Kappa	0.7917	0.7428	0.7273	0.5171	0.5471
Precision	0.8929	0.8750	0.8706	0.7952	0.8125
Recall	0.9868	0.9589	0.9737	0.9041	0.9873
F-score	0.9375	0.9150	0.9193	0.8462	0.8914
**vCPU = 8 RAM = 32 GB**					
Accuracy	0.9429	0.9516	0.9670	**0.9780**	0.9758
Kappa	0.7567	0.7381	0.6297	0.5214	0.5190
Precision	0.8764	0.8471	0.7976	0.7692	0.7857
Recall	1.0000	1.0000	0.9710	0.9722	0.9167
F-score	0.9341	0.9172	0.8758	0.8589	0.8462
**vCPU = 16 RAM = 64 GB**					
Accuracy	0.9363	0.9538	0.9692	0.9758	**0.9824**
Kappa	0.8786	0.7162	0.4736	0.5015	0.3889
Precision	0.9259	0.8837	0.7045	0.7143	0.7528
Recall	1.0000	0.9620	0.9688	0.9524	0.9054
F-score	0.9615	0.9212	0.8158	0.8163	0.8221
**vCPU = 36 RAM = 60 GB**					
Accuracy	0.9385	0.9582	0.9714	0.9802	**0.9868**
Kappa	0.8064	0.6769	0.6341	0.4734	0.4302
Precision	0.8507	0.8049	0.7895	0.8068	0.7326
Recall	0.9828	0.9851	0.9375	0.9103	0.9130
F-score	0.9120	0.8859	0.8572	0.8554	0.8129

## Data Availability

Publicly available dataset was analyzed in this study. This data can be found here: https://archive.ics.uci.edu/ml/machine-learning-databases/breast-cancer-wisconsin/.
